# Biomarking Trait Resilience With Salivary Cortisol in Chinese Undergraduates

**DOI:** 10.3389/fpsyg.2020.536510

**Published:** 2020-10-26

**Authors:** Julian C. L. Lai, Monique O. Y. Leung, Daryl Y. H. Lee, Yun Wah Lam, Karsten Berning

**Affiliations:** ^1^Psychophysiology Laboratory, Department of Social and Behavioural Sciences, City University of Hong Kong, Kowloon, Hong Kong; ^2^Department of Chemistry, City University of Hong Kong, Kowloon, Hong Kong

**Keywords:** resilience, Brief Resilience Scale, salivary cortisol, Chinese undergraduates, hypothalamic-pituitary-adrenocortical axis

## Abstract

This study aimed to examine the relationship between trait resilience and salivary cortisol in a group of Chinese undergraduates. The Chinese versions of the Brief Resilience Scale and a measure of optimism, the revised Life Orientation Test were administered to 49 Chinese undergraduates who provided self-collected saliva samples six times per day (immediately after waking; 0.5, 3, 6, and 12 h thereafter; and at bedtime) over 3 consecutive weekdays. The cortisol data were aggregated across the 3 days to examine the association between resilience and components of the diurnal rhythm of cortisol using multiple regression. The results showed that higher resilience was associated with a stronger cortisol response to awakening and a steeper diurnal decline in cortisol from waking to bedtime. Resilience was positively associated with cortisol output over the course of the day but this relationship was not significant (*p* = 0.065). This pattern of diurnal rhythm is consistent with that typically observed in better adjusted individuals. Generated by an intensive protocol with compliance objectively monitored, these findings clearly indicate the important role of the hypothalamic-pituitary-adrenocortical axis in health and adjustment and contribute to the growing literature on resilience and cortisol in humans.

## Introduction

The past two decades have witnessed a proliferation of research on resilience and the associated health implications in different populations (Norris et al., 2009). Resilience was first depicted by researchers in child and adolescent development as a factor enabling children to develop into well-adjusted adults amid adversity. Research on this construct has since been extended to adult populations (Masten and Reed, 2002) and has more recently encompassed optimal functioning (Schetter and Dolbier, 2011). Although the concept of resilience has more than one definition in the literature (Ahern et al., 2006), it is now widely appreciated that this concept refers to the ability to emerge or bounce back from stress or to remain healthy in the face of adversity (Smith et al., 2008; Wu et al., 2013; Lai and Yue, 2014). Metaphorically, it refers to the tendency to “bend but not break” in the face of stress (Karatsoreos and McEwen, 2013). Recent research has illuminated the neurobiology of resilience using animal models (reviewed by Cathomas et al., 2019), which was subsequently extended to humans (Frodl and O’Keane, 2013). It is now widely appreciated that exposure to adversities in early stages of development plays a crucial role in programming the cortisol response to stress in adulthood *via* the hypothalamic-pituitary-adrenocortical (HPA) axis (Boersma and Tamashiro, 2015; King, 2016). In addition to early adversities, ongoing life stress also affects the regulation of cortisol levels by increasing the allostatic load in vulnerable individuals (e.g., Spencer-Segal and Akil, 2019).

Cortisol is the hormonal end-product of HPA axis. Dysregulation in the diurnal cortisol output or rhythm has been associated with various pathological conditions such as depression (Weber et al., 2000; Bhagwagar et al., 2003), post-traumatic stress disorder (Labonté et al., 2014), and inflammatory diseases (Nijm et al., 2007). The circadian rhythm of cortisol is characterized by a substantial increase in the cortisol level within the first hour after waking, peaking at about 30–45 min post-waking (Clow et al., 2004). This is followed by a gradual decline over the course of the day until the nadir is reached around midnight, after which the cortisol level gradually increases during nocturnal sleep until waking the next morning (Van Cauter et al., 1996). Given the large volume of evidence demonstrating a positive association of resilience with better health (reviewed by Hu et al., 2015; Ávila et al., 2017) and the close association between diurnal cortisol rhythms and health outcomes (reviewed by Caulfield and Cavigelli, 2020), it is likely that resilience is marked by the same diurnal cortisol rhythm associated with better health.

Despite the centrality of cortisol regulation in the biological basis of resilience, research on the association between resilience and the diurnal rhythms of cortisol in humans is limited, and the findings preclude concrete conclusions. In particular, higher resilience was reported to be associated with lower waking levels and lower overall levels in the 60-min post-awakening period among 67 parents of people with autism spectrum disorder (ASD; Ruiz-Robledillo et al., 2014). In line with this finding, resilient Chinese servicemen exposed to experimental sleep deprivation exhibited an attenuated increase in serum cortisol at 8 AM in the morning compared to their non-resilient peers (Sun et al., 2014). In a study with 645 Chinese children (mean age = 10.67 years) with parents infected with HIV (Chi et al., 2015), higher resilience was found to be associated with higher waking and diurnal levels of salivary cortisol, and a steeper diurnal slope. However, resilience had no relationship with cortisol awakening response (CAR). In another study with a community sample of 32 participants, resilience was found to be unrelated to waking cortisol levels (Petros et al., 2013).

Heterogeneity in the design of the reviewed studies and mixed findings make integration particularly challenging. Different protocols were adopted to collect saliva samples to examine the diurnal rhythm of cortisol. Saliva samples were collected four times over 2 days in post-awakening period (Ruiz-Robledillo et al., 2014) but were collected four times from waking to bedtime over 3 days in Chi et al. (2015). On the other hand, only one single saliva sample was collected in the other two studies (Petros et al., 2013; Sun et al., 2014). In addition, the instrument used to measure the construct of resilience also vary across studies (Connor and Davidson Resilience Scale, Petros et al., 2013; Chi et al., 2015; Brief Resilient Coping Scale, Ruiz-Robledillo et al., 2014; the military personnel mental resilience scale, Sun et al., 2014). The interpretability of findings on diurnal cortisol levels is subject to methodological limitations because compliance was not monitored objectively using proper devices in all studies and only one single saliva samples was collected on one single day in Petros et al. (2013) and Sun et al. (2014). Reliable monitoring of saliva sampling times is crucial for accurate assessment of changes in cortisol levels during the post-awakening period, during which cortisol levels are most volatile (Stalder et al., 2016). The difference in the operationalization of resilience across these four studies further intensifies the challenge of integration.

In view of the limitations of the aforementioned studies and the scarcity of data regarding the association between resilience and cortisol, the present study was designed to re-examine the association between trait resilience and diurnal cortisol rhythms. We addressed the methodological issues using an intensive protocol that monitored compliance with a proper electronic device, and the Brief Resilience Scale (BRS), a valid measure of resilience more applicable than other measures to Asian respondents in general (Malaysians: Amat et al., 2014; Indians: Kumar and Dixit, 2014) and Chinese respondents in particular (Lai and Yue, 2014). Studying resilience in a sample of college students is appropriate because recent evidence has shown that a higher education environment is challenging, as indicated by the prevalence of psychiatric disturbances (e.g., Blanco et al., 2008), as well as growth-stimulating (Lai et al., 2001). This moderately stressful environment provides a suitable context to examine the emergence of resilience.

The association between resilience and cortisol has been studied in prior studies with respect to the CAR, diurnal slope (DS), and diurnal output over the course of the day operationalized as the area under the curve with reference to ground (AUC_G_; Pruessner et al., 2003). These three indices provide complementary information about the diurnal rhythm of cortisol. The CAR refers to the rise in cortisol from waking to the peak at 30–45 min after waking. Past research has shown that the average increase in the waking cortisol value is between 50 and 160% (Clow et al., 2004). The CAR is regarded as “the first and the largest ultradian episode of the day” (Evans et al., 2019, p. 250) and marks the beginning of circadian activity (Lightman and Conway-Campbell, 2010). This component of the cortisol diurnal rhythm is positively associated with better adjustment in elders (Evans et al., 2012; Lai et al., 2012) and younger adults (Powell and Schlotz, 2012; Marin et al., 2019). A stronger CAR is also associated with younger age (Heaney et al., 2012). On the other hand, attenuation of the CAR is associated with several undesirable conditions such as poor general health (Lasikiewicz et al., 2008), burnout (Oosterholt et al., 2015), and subclinical depression (Dedovic and Ngiam, 2015). The DS is commonly operationalized as the change in cortisol per unit of time from waking to bedtime or late evening (Kraemer et al., 2006). A flatter DS has been shown to be associated with poorer general health in two recent reviews (Adam et al., 2017; Caulfield and Cavigelli, 2020), whereas a steeper slope is associated with higher resilience in children (e.g., Chi et al., 2015). The CAR can be considered to indicate the effectiveness of cortisol activation, whereas the DS can be regarded as the effectiveness of deactivation: the decline of cortisol from waking to late evening. A stronger CAR and a steeper DS (Lai et al., 2012), and a lower diurnal level (Lai et al., 2017) have been observed in better adjusted Chinese seniors by the first author and associates in separate studies. Lai and Lee (2018) suggested that this pattern could be a central neuroendocrine feature of better health in elders.

The area under the curve with reference to ground refers to the total secretion of cortisol over the course of a day. An accentuated AUC_G_ has been observed in many pathological conditions such as chronic burnout (Melamed et al., 1999), depression (Belanoff et al., 2002), and chronic stress (Bauer et al., 2000). In healthy populations, a lower AUC_G_ is associated with lower loneliness in college students (e.g., Lai et al., 2019) and better adjustment (Chin et al., 2017; Lai et al., 2017). A recent study in healthy participants has demonstrated that a lower AUC_G_ is associated with a lower risk of experimentally induced upper respiratory infection (Janicki-Deverts et al., 2016).

To recapitulate, the goal of present study was to examine the association between resilience and the diurnal rhythm of salivary cortisol using an intensive protocol with compliance objectively monitored, and a measure of resilience demonstrated to be appropriate for Chinese populations. On the basis of findings reviewed earlier, we hypothesized that higher resilience should be associated with a diurnal profile of cortisol characterizing better health or adjustment. This hypothesis was tested by examining the associations of resilience with CAR, DS, and AUC_G_ separately. We expected that higher resilience would be associated with a steeper DS because better health (e.g., Adam et al., 2017) and higher resilience (e.g., Chi et al., 2015) have been found to be associated with a steeper DS. With regard to CAR and AUC_G_, we were not able to formulate specific predictions concerning their relationships with resilience due to the lack of converging evidence showing an association of these two indices with resilience and better health.

## Materials and Methods

### Participants

Forty-nine Hong Kong Chinese students (*n* = 28 females; mean age = 20.92; *SD* = 1.94; range = 18–29) recruited from an undergraduate introductory psychology class at a university in Hong Kong participated in the study voluntarily. The majority (61.2%) were in their second year of undergraduate study. The participants had no known diagnosis of psychiatric disorders or cardiovascular diseases, and were not currently on medication that could potentially affect cortisol levels. They did not smoke habitually. There was no oral contraceptive user among the female participants. Course credits and cinema vouchers were given in return for participation. This study was approved by the Human Subject Ethics Sub-Committee of the College of Liberal Arts and Social Sciences of the City University of Hong Kong. Informed consent was obtained from all participants. Initially, 54 participants were recruited, five of whom were excluded because they either did not meet the inclusion criteria or failed to provide sufficient saliva samples for the cortisol analysis.

### Procedure

This study followed the procedure used by Lai et al. (2019) in a prior study with Chinese undergraduates. Participants were given in the briefing session detailed description of the saliva sampling procedure and all the instructions and materials they needed for the study, including saliva sampling tubes (Salivette) and the MEMS TrackCaps (WestRock, Sion, Switzerland) for monitoring the timing of saliva sampling. Over 3 consecutive weekdays, participants were required to collect by themselves six saliva samples each day at 0, 0.5, 3, 6, and 12 h after waking and at bedtime, using the Salivettes. Participants were asked to refrain from exercise, smoking, brushing teeth, eating, and consuming beverages containing alcohol or caffeine before the collection of the first two saliva samples and 1 h before the collection of the remaining four samples. A window of 5 min was adopted for the waking and 30-min saliva samples, and a 30-min window for subsequent samples (Stalder et al., 2016). Participants were required to put down the waking time of each day and the time at each saliva sample to be collected and were collected subsequently on a diary log. At the end of the briefing session, participants filled out a questionnaire containing scales measuring resilience and optimism, and items for collecting health and demographic information relevant to the present study. Participants were told to store their saliva samples in their home freezers until returning them to the laboratory no later than 7 days after saliva sampling had completed. The returned samples were then stored in the laboratory at −20°C until thawed for biochemical analysis.

### Measures

#### Resilience

The participants’ resilience was measured using an adapted version of the BRS (Lai and Yue, 2014), which was originally developed by Smith et al. (2008) to measure the ability to bounce back from stress. The scale has been chosen because of its relevance to non-traumatized populations such as university students (Lai and Yue, 2014), sound psychometric properties (Windle et al., 2011), and applicability to studies with Asian populations (e.g., Malaysians: Amat et al., 2014; Indians: Kumar and Dixit, 2014; and Chinese: Lai and Yue, 2014). The scale consists of three items worded positively and three items worded negatively. To complete the scale, the participants were asked to indicate the extent to which they agreed with each of the six items according to a 5-point rating scale (1 = strongly disagree; 5 = strongly agree). The BRS was scored by reverse coding the negatively worded items and calculating the sum of all six items. Higher scores indicate higher resilience. The scale exhibited acceptable internal consistency in the present sample, with a Cronbach’s alpha of 0.77.

#### Optimism

This was measured by the Chinese version of the revised Life Orientation Test, which was validated in different Chinese populations by the first author and his associates (e.g., Lai, 2009; Lai and Mak, 2009). The scale consists of three positively worded items and three negatively worded items. Each item was assessed by using a 5-point rating scale (1 = strongly disagree; 5 = strongly agree). The sum of the six items was computed by adding the ratings of the positive and reversed negative items, with higher total scores represent higher levels of optimism. It has been shown to be internally consistent in studies with Chinese populations and showed a Cronbach’s alpha of 0.86 in the present sample. Optimism was mainly treated as a covariate because of its conceptual connection with the construct of resilience (Lai and Yue, 2014) and its association with salivary cortisol in Chinese populations (Lai et al., 2005).

### Cortisol Assays

Cortisol concentrations were analyzed using an enzyme-linked immunosorbent assay (Enzo Life Sciences, Inc.) similar to those used in prior studies in Hong Kong Chinese participants (e.g., Lai et al., 2019). The assays were performed in the laboratory of the Chemistry Department at the City University of Hong Kong. The stored saliva samples were thawed and centrifuged at 3500 rpm for 15 min at room temperature, and the obtained clear supernatants were used for analysis. The sensitivity of the assays was 0.2 nmol/L. Intra- and inter-assay coefficients of variation were lower than 12%, which is comparable to similar assays used in prior studies in Hong Kong Chinese participants (Lai et al., 2018, 2019).

### Statistical Analyses

Relationships between key variables of the present study, and that between cortisol levels at each sampling time across the 3 days were examined using product-moment correlations. ANOVA with repeated measures was used to analyze the changes of cortisol levels each day over the 3 days. The Greenhouse-Geisser correction was adopted for violation of the sphericity assumption. With respect to the relationships between resilience and the three cortisol parameters: CAR, DS, and AUC_G_, cortisol data were aggregated over the 3 days. The DS was computed as the decrease in the cortisol level per hour from waking to bedtime for each individual: [(level at bedtime – waking level)/the specific time interval from waking to bedtime for each participant; e.g., Lai et al., 2019]. The CAR was operationalized as the increase in the cortisol level from waking to 30 min after waking and computed by the AUC_I_ formula proposed by Pruessner et al. (2003, p. 920, formula 5). The AUC_G_ was derived by applying the formula proposed by Pruessner et al. (2003, p. 919, formula 1); all six samples over the course of a day were used in the computation for the AUC_G_. Time intervals between sampling times for the computations were based on the number of hours from waking. Daily indices were computed using the aforementioned formulae and the means across 3 days (CAR_M_, AUC_GM_, and DS_M_) were then derived to be used as the criterion variables in subsequent regression analyses. The computation of DS, AUC_I_, and AUC_G_ for each individual was based on objective timings of saliva collection from each participant, which was not possible without using the MEMS TrackCaps or equivalent devices. Although waking times were not monitored objectively, participants were required to provide their waking times on each day. Discrepancies between self-reported waking times and the time stamps associated with the first saliva samples on each day were used to estimate the reliability of the timings of the first samples on each day. The mean difference was 1.7 min (*SD* = 2.12), 2.31 min (*SD* = 3.96), and 1.63 min (*SD* = 1.90), on the first, second, and third day, respectively. The small magnitudes of the mean discrepancies confer assurance that the CAR examined in our study was not based on an unreliable estimate of the peak level of cortisol.

Hierarchical multiple regression analyses were applied to uncover the association between resilience (predictor variable) and each of the three cortisol parameters (criterion variables). Gender, age, mean waking time of the 3 days, compliance, and optimism were treated as control variables and entered before resilience in the regression analyses. To control for the effect of non-compliance on cortisol parameters, three variables were created on each of the 3 days, with compliance coded as 0 and non-compliance coded as 1. Participants were then divided into four groups according to the sum of non-compliance across the 3 days, with scores ranging from 0 to 3 (0 meaning that all saliva samples were collected at the scheduled times over the 3 days and 3 meaning that at least one saliva sample was not collected at the scheduled time on each of the 3 days). Cortisol data were not normalized for the computations of the three cortisol indices and multiple regression analyses because normality is not an important issue particularly for the latter (Licht, 1997). The assumptions of normality in residuals, homoscedasticity, and multicollinearity are more relevant to multiple regression. None of these three conditions were shown to be violated in the regression analyses according to normality plots, residuals plots, and tolerance indices generated by SPSS.

## Results

### Cortisol Data

Non-compliance in participants, which was defined as the failure to self-collect saliva samples at one or more scheduled sampling times. Specifically, a sample was considered as non-compliant if it was collected out of the designated window of collection: 5 min for the waking and 30-min sample; 30 min for the next three samples; and no window for the bedtime sample. The percentages of non-compliant participants were 28.6, 36.7, and 51% on the first, second, and third day, respectively. Cortisol data without a time stamp or beyond the detection limits of the assays (79 samples), were treated as missing and imputed using the expectation-maximization method (IBM SPSS 26) and then winsorized at the low end to 0.2 nmol/L (Lai et al., 2018, 2019).

**Figure 1:**
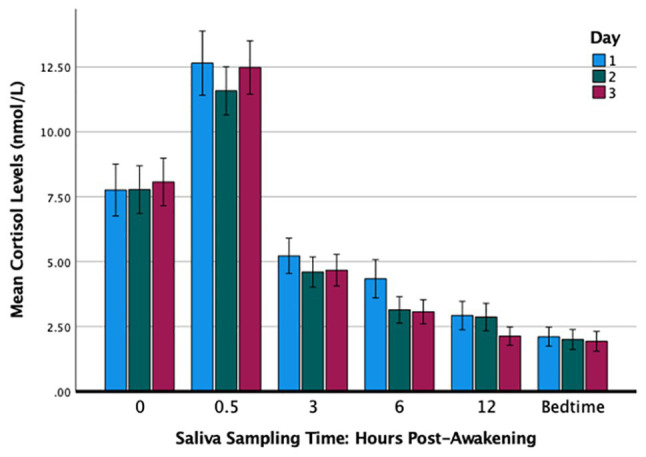
Changes in cortisol levels over time across 3 consecutive days. Error bars: +/−1 SE.

Table 1 summarizes the cortisol levels over the 3 days. The results of a 3 × 6 analysis of variance with repeated measures (day by sampling times) and adjustments using the Greenhouse-Geisser correction showed that the main effect of day was not significant [*F*(1.70,81.66) = 1.17, *p* = 0.308, partial *η*^2^ = 0.02] and nor was the interaction effect [*F*(4.65,223.31) = 1.11, *p* = 0.354, partial *η*^2^ = 0.02]. The effect of sampling times was significant in that the cortisol levels varied significantly throughout the day [*F*(1.61,77.29) = 118.19, *p* < 0.001, partial *η*^2^ = 0.71] and exhibited a typical diurnal rhythm with the peak at 30 min post-waking and the nadir at bedtime (Figure 1). The cortisol levels over the 3 days were significantly and positively correlated at each of the six sampling times: waking (*r*s ranged from 0.75 to 0.81), 30 min (0.54 to 0.78), 3 h (0.45 to 0.70), 6 h (0.45 to 0.69), 12 h (0.69 to 0.81), and bedtime (0.66 to 0.84).

**Table 1:** Mean (SEM) of cortisol levels (nmol/L) across 3 days.

Day	Saliva sampling times (hours post-awakening)					
	Waking	0.5 h	3 h	6 h	12 h	Bedtime
1	7.76 (1.00)	12.65 (1.23)	5.23 (0.68)	4.34 (0.74)	2.93 (0.55)	2.11 (0.36)
2	7.78 (0.92)	11.59 (0.93)	4.60 (0.58)	3.14 (0.51)	2.87 (0.53)	2.00 (0.38)
3	8.07 (0.92)	12.48 (1.03)	4.67 (0.61)	3.07 (0.46)	2.13 (0.35)	1.93 (0.38)
Combined	7.87 (0.87)	12.24 (0.93)	4.83 (0.53)	3.52 (0.48)	2.64 (0.44)	2.02 (0.35)

### Resilience and Cortisol

The mean values (standard deviations) of the three cortisol indices were as follows: AUC_GM_ = 68.41 (55.66), DS_M_ = −0.37 (0.30), and CAR_M_ = 1.20 (0.76). This implies that cortisol increased at a rate of 8.33 nmol/L per hour within the first 30 min after waking and dropped at a rate of 0.37 nmol/L per hour from waking to bedtime in the participants. Table 2 summarizes the product-moment correlation coefficients between the key variables examined in the present study. Gender, age, waking time, compliance, and optimism showed no correlation with the three indices of cortisol rhythm. BRS scores were correlated positively with the CAR_M_ (*r* = 0.33, *p* = 0.021), and negatively with DS_M_ (*r* = −0.36, *p* = 0.011) but not with the AUC_GM_ (*r* = 0.24, *p* = 0.093). The significant correlation between gender and age was attributable to a female participant whose age was more than 3 standard deviations above the mean.

**Table 2:** Product-moment correlations between key variables and cortisol indices.

Variable	Age	Waking time	Compliance	Optimism	Resilience	CAR_M_	DS_M_	AUC_GM_
Gender	0.44^**^	0.07	−0.09	0.02	0.05	0.03	0.13	−0.06
Age		0.13	−0.05	−0.03	−0.21	−0.05	0.05	−0.03
Waking time			0.10	−0.24	−0.15	−0.21	−0.10	0.17
Compliance				−0.02	−0.03	0.08	−0.07	0.02
Optimism					−0.06	0.04	0.24	−0.20
Resilience						0.33^*^	−0.36^*^	0.24
CAR_M_							0.10	0.09
DS_M_								−0.57^**^
Mean	20.92	09:16	1.16	19.35	19.90	4.37	−0.37	68.41
*SD*	1.94	1 h 16 min	0.75	4.54	3.26	2.67	0.30	55.66

CAR_M_, cortisol awakening response; DS_M_, diurnal slope; AUC_GM_, area under the curve with reference to ground; gender: male = 0, female = 1.

* *p* < 0.05;

** *p* < 0.01.

The associations between resilience and cortisol were further examined using multiple regression analysis, while controlling for the effects of gender, age, waking time, compliance, and optimism. Resilience and covariates were entered hierarchically in regression equations in the following order: gender (male = 0, female = 1), age, waking time, and compliance over 3 days were all entered in block 1, optimism in block 2, and resilience in block 3.

Table 3 summarizes the results of the analysis. Higher resilience was significantly associated with a stronger CAR_M_ (*B* = 0.07, 95% CI = 0.001, 0.410) and a steeper DS_M_ (*B* = −0.04, 95% CI = −0.065, −0.012) but resilience had no significant relationship with the diurnal output of cortisol or AUC_GM_ (*B* = 4.94, 95% CI = −0.312, 10.193). This pattern of findings implies that higher resilience is associated with more effective activation and deactivation of the HPA axis, which is in line with the diurnal rhythm characterizing better heath in studies mentioned earlier (e.g., DS, Adam et al., 2017; CAR, Lasikiewicz et al., 2008).

**Table 3:** Full linear regression models predicting cortisol indices.

Predictor	CAR_M_			DS_M_			AUC_GM_		
	*B*	*t*	*p*	*B*	*t*	*p*	*B*	*t*	*p*
Gender	0.02	0.07	0.943	0.11	1.22	0.231	−11.47	−0.64	0.529
Age	0.02	0.22	0.824	−0.01	−0.61	0.547	1.43	0.30	0.767
Waking time	−0.10	−1.05	0.299	−0.05	−0.73	0.467	8.37	1.25	0.220
Compliance	0.11	0.76	0.452	−0.02	−0.39	0.697	−0.33	−0.03	0.976
Optimism	0.004	0.15	0.885	0.01	1.27	0.209	−1.65	−0.91	0.371
Resilience	0.07	2.06	0.046	−0.04	−2.76	0.009	4.94	1.90	0.065

CAR_M_, cortisol awakening response; DS_M_, diurnal slope; AUC_GM_, area under the curve with reference to ground; gender: male = 0, female = 1.

## Discussion

Our findings demonstrated that higher resilience was associated with both a stronger CAR and a steeper diurnal decline in a young and healthy sample of undergraduates. An accentuated CAR can be taken to indicate effective coping with life stress (Powell and Schlotz, 2012), a steeper DS more effective cortisol regulation (Adam et al., 2017). Characterized by effective activation and deactivation of the HPA axis, this cortisol rhythm provides a neuroendocrine explanation for the well-documented positive association between resilience and general health (Hu et al., 2015; Ávila et al., 2017). This cortisol rhythm has been observed in better adjusted Chinese seniors (Lai et al., 2012) and better adjusted middle-aged participants in a study with a United States national sample (Friedman et al., 2012). The association between this cortisol rhythm and better health is also confirmed in a recent review (Caulfield and Cavigelli, 2020). However, resilience exhibited a positive but nonsignificant relationship with diurnal cortisol output in the present study, which is not consistent with the association between an accentuated AUC_G_ and poor health reported in prior studies (e.g., Janicki-Deverts et al., 2016). The reasons for this nonsignificant finding are not immediately apparent, but it could be attributed to the small sample size of our study and a relatively small effect size of the relationship. On the other hand, AUC_G_ may be associated with resilience differentially in severely stressed or traumatized samples (e.g., enhanced AUC_G_ in more resilient children with parents infected with HIV, Chi et al., 2015) vs. non-traumatized populations such as healthy undergraduates in our study. AUC_G_ could depend more on factors such as age and severity of stress exposure or a combined of these factors compared to the other two cortisol indices. However, these contentions are purely speculative and the issues should be addressed empirically in future studies.

Taken together, our findings can be attributed to methodological merits which may have important implications for future research. First, the use of the BRS in our study illustrates the importance of selecting a measure of resilience appropriate for the objective and the target population of a study. As mentioned earlier, the BRS is not only a conceptually valid measure of resilience but also more applicable to Chinese and other Asian populations. Most importantly, the BRS assesses the perceived efficacy to emerge from stress and mediates the effects of “conventional” personal resources such as self-esteem and optimism on physical health in Chinese undergraduates (Lai and Yue, 2014). This may explain why our findings clearly indicate that higher resilience is associated with a more adaptive diurnal cortisol rhythm, which has not been reported in prior studies in Chinese participants (Sun et al., 2014; Chi et al., 2015). Although, consistent with our findings, higher resilience has been reported to be associated with a steeper DS by Chi et al. (2015), resilience was positively associated with waking cortisol but not CAR. The health implication of an enhanced waking cortisol level is unclear because resilience has also been shown to be associated with a lower waking level in parents of people with ASD (Ruiz-Robledillo et al., 2014). Our preliminary findings may provide useful guidelines for the selection of appropriate measure of resilience in future research.

Second, we used individualized timings recorded by the MEMS TrackCaps in the computation of the three cortisol indices, which has rarely been carried out in prior studies examining resilience and cortisol. This has an important implication for the computation of cortisol indices taking time intervals between saliva samples into account, such as the formulae proposed by Pruessner et al. (2003). For example, in the computation of DS, an equal drop in cortisol level could lead to different DS for two individuals who collected the wake-up sample at about the same time but one went to bed much later. This important information will be missing in the computations of cortisol indices without an objectively generated time stamp of each saliva sample. Our approach enhances the variation in cortisol data to facilitate more accurate assessment of the diurnal rhythm, and is thus recommendable for future research.

Despite the significance of the present findings, their application has several limitations. The cross-sectional design of our study precludes the drawing of any causal connections between resilience and cortisol. Longitudinal or intervention studies are needed to address the issue of causality. We were not able to examine the potentially protective or buffering effect of resilience in the context of stress because transient or chronic stress has not been examined. Further research is warranted to illuminate this important issue, preferably using a longitudinal design. The control of confounding variables is another area that could be improved because health behaviors such as total sleep hours (e.g., Vargas and Lopez-Duran, 2014) that has shown to affect cortisol, have not been controlled. The sample size of *N* = 49 may not be optimal for the regression analyses, but the major assumptions of linear regression have not been violated. This may hopefully provide a reasonable assurance of the reliability of our findings (Licht, 1997). However, further research on the relationship between resilience and cortisol should use a larger sample size to enhance reproducibility, especially regarding the relationship between resilience and AUC_G_. Researchers may also consider extending the applicability of present findings to different age groups or populations.

Although we did not find any significant correlations between non-compliance and cortisol, the proportion of non-compliant participants increased over the 3 days from 28.6% on day 1 to 51% on day 3. This raises at least two issues. First, methods to reduce non-compliance should be implemented in future research, although protocols that require participants to collect saliva samples at different times synchronized to their individual waking times are challenging for participants. Second, if non-compliance increases over sampling days because of the increasing burden on participants, researchers should develop a protocol with the optimal numbers of sampling days and times per day to best suit the purposes of their research and avoid overburdening participants. Hellhammer et al. (2007) found that to reliably assess the contribution of trait factors to the CAR, data of 6 days are required because the influence of state or situational factors is greater for data of fewer days. On the other hand, reliable assessment of the DS requires at least two samples (at waking and evening) per day for 3 days (Kraemer et al., 2006). These findings provide an empirical foundation for future studies to minimize the burden on participants and optimize the cost of research.

## Conclusion

Human research on resilience has focused on the mental health correlates of the construct while paying insufficient attention to physical health outcomes. The present study aimed to fill this knowledge gap by examining the association between resilience and a well-established biomarker of health, cortisol. Using an intensive protocol involving the collection of six saliva samples per day over 3 consecutive weekdays with objective monitoring of saliva sampling times, we showed that in healthy undergraduates, higher resilience is associated with an enhanced CAR and a steeper DS, both of which are central features of the cortisol rhythm observed in healthier or better adjusted individuals. Our findings contribute to an area of growing importance in the literature and will hopefully stimulate more research in this direction. To better understand the mechanisms that translate resilience into better health outcomes, future research should pay increased attention to the methodological issues discussed earlier, and to biological and psychosocial factors contributing to a better understanding of the ontogenesis of the resilient phenotype.

## Data Availability Statement

The original contributions presented in the study are included in the article/supplementary material, further inquiries can be directed to the corresponding author.

## Ethics Statement

The studies involving human participants were reviewed and approved by Human Subject Ethics Sub-Committee of the College of Liberal Arts and Social Sciences of the City University of Hong Kong. The patients/participants provided their written informed consent to participate in this study.

## Author Contributions

JL was responsible for the design of the study, data analysis, interpretation of data, and the write-up of the manuscript. ML, YL, and KB were responsible for the acquisition of data. DL contributed to interpretation of data and the write-up of the manuscript. All authors read and approved the submitted version of the manuscript.

### Conflict of Interest

The authors declare that the research was conducted in the absence of any commercial or financial relationships that could be construed as a potential conflict of interest.
